# Prevalence and Risk Factors of Gang Membership in a Brazilian Birth Cohort

**DOI:** 10.1001/jamanetworkopen.2024.40393

**Published:** 2024-10-21

**Authors:** Andreas Bauer, Rafaela Costa Martins, Gemma Hammerton, Hugo Gomes, Helen Gonçalves, Ana M. B. Menezes, Fernando C. Wehrmeister, Joseph Murray

**Affiliations:** 1 Postgraduate Program in Epidemiology, Federal University of Pelotas, Pelotas, Brazil; 2 Human Development and Violence Research Centre (DOVE), Federal University of Pelotas, Pelotas, Brazil; 3 Centre for Academic Mental Health, Population Health Sciences, Bristol Medical School, University of Bristol, Bristol, UK; 4 Medical Research Council Integrative Epidemiology Unit, Population Health Sciences, Bristol Medical School, University of Bristol, Bristol, UK; 5 Epidemiology Research Unit, Institute of Public Health, University of Porto, Porto, Portugal

## Abstract

**Importance:**

There is no longitudinal evidence on risk factors for gang membership in low- and middle-income countries, despite organized crime groups posing major challenges, including high homicide rates in Latin America. Furthermore, adverse childhood experiences (ACEs) have been largely overlooked in gang-related research worldwide.

**Objectives:**

To examine the associations of ACEs up to 15 years of age with past-year gang membership at 18 years of age and to compare crime and criminal justice involvement between gang members and non–gang members.

**Design, Setting, and Participants:**

This cohort study assessed children from the 1993 Pelotas (Brazil) Birth Cohort—an ongoing population-based, prospective study. Assessments were undertaken perinatally (1993) and when the children were ages 11 (2004), 15 (2008), 18 (2011), and 22 (2015) years. All children born in 1993 were eligible (N = 5265), and 5249 (99.7%) were enrolled at birth. The study sample (N = 3794 [72.1%]) included those with complete data on ACEs. Data analyses were conducted from February to August 2024.

**Exposures:**

Twelve ACEs were assessed up to 15 years of age via child self-report and/or maternal report, including physical neglect, physical abuse, emotional abuse, sexual abuse, domestic violence, maternal mental illness, parental divorce, ever being separated from parents, parental death, poverty, discrimination, and neighborhood fear. These experiences were examined using a single adversity approach, cumulative risk, and latent classes.

**Main Outcomes and Measures:**

The main outcome was past-year gang membership at 18 years of age, assessed via self-report and analyzed using multivariate imputation.

**Results:**

Of 3794 participants, 1964 (51.8%) were female and 1830 (48.2%) were male, and 703 (18.5%) were Black, 2922 (77.0%) were White, and 169 (4.5%) were coded as “other” race or ethnicity (no additional details are available to further disaggregate the other category). On the basis of the imputed data, 1.6% (SE, 0.2 percentage points) of participants reported gang membership at 18 years of age. Physical abuse (odds ratio [OR], 2.76; 95% CI, 1.27-5.98), emotional abuse (OR, 2.76; 95% CI, 1.51-5.02), domestic violence (OR, 3.39; 95% CI, 1.77-6.48), parental divorce (OR, 2.04; 95% CI, 1.17-3.54), and separation from parents (OR, 3.13; 95% CI, 1.54-6.37) were associated with an increased risk of gang membership. A dose-response association was observed, with 4 or more ACEs increasing the risk (OR, 8.86; 95% CI, 2.24-35.08). In latent class analysis, the class with child maltreatment and household challenges was associated with a higher risk of gang membership than the low-adversities class (OR, 7.10; 95% CI, 2.37-21.28). There was no robust evidence that children exposed to household challenges and social risks were at increased risk of gang membership (OR, 2.28; 95% CI, 0.46-11.25).

**Conclusions and Relevance:**

In this prospective cohort study, ACEs, particularly child maltreatment and family conflict, were associated with gang involvement when examined individually, cumulatively, and as clusters in a high-crime environment in Brazil. These findings underscore the value of integrating the ACE framework into gang-related research and the potential to reduce gang-related crime by reducing ACEs.

## Key Points

**Question:**

Are adverse childhood experiences (ACEs) associated with increased risk of gang membership in a low- and middle-income country?

**Findings:**

Among 3794 participants in a population-based birth cohort study in Brazil, 1.6% reported gang membership. Adverse childhood experiences (particularly those related to child maltreatment and family conflict) were associated with later gang involvement when examined individually, cumulatively, and as clusters.

**Meaning:**

These findings suggest that exposure to ACEs is associated with increased risk of gang involvement, underscoring the importance of integrating the ACE framework into gang-related research and highlighting the potential to prevent gang-related crime by reducing ACEs.

## Teaser

This cohort study of a 1993 Brazilian birth cohort examines the associations between adverse childhood experiences and gang membership.

## Introduction

Organized crime groups pose a serious social, justice, and public health problem, accounting for a substantial proportion of homicides—22% worldwide and 50% in North and South America.^1^ Gang operations, largely fueled by a staggering US $2.1 trillion drug trafficking trade (equivalent to 3.6% of the global gross domestic product),^2^ severely undermine local economies, institutions, and social life.^3^ Organized gangs may replace, collaborate with, or compete against state authorities, creating a perilous blend of criminal governance, corruption, and pervasive violence.^4^ As a major setting in the production and distribution of illicit drugs, especially cocaine,^5^ Latin America represents a critical context for gang-related research.

Brazil has a homicide rate of 22.3 per 100 000 people,^6^ with interpersonal violence being the leading cause of death among children and adolescents.^7^ During the past 2 decades, Brazil witnessed a marked increase in its prison population, almost quadrupling from 230 000 in 2000 to 830 000 in 2022 (410 prisoners per 100 000 people).^8^ This era of mass incarceration has likely contributed to the emergence and growth of organized crime groups, commonly referred to as prison gangs in the Brazilian context.^9^ Although current estimates identify 53 gangs across Brazil,^10^ this figure likely falls short of the true number when smaller groups confined to particular low-income neighborhoods, or *favelas*, are considered.^11^

Current evidence from high-income countries, mainly the US and European countries, shows that gang membership is driven by risk factors across multiple domains, including individual, family, peer, school, and community factors.^12,13,14^ However, social and cultural contexts shape gang formation,^15^ and the generalizability of these findings to regions such as Latin America, where gangs are a major challenge, with underpinnings in highly dysfunctional criminal justice systems, deep inequality, and enormous drug trade challenges, is unclear. Higginson et al^16^ conducted a systematic review of evidence on risk and protective factors for gang membership in low- and middle-income countries. The 9 studies that were identified (from searches in 7 languages) revealed findings largely consistent with those from high-income countries. However, all studies were cross-sectional, and only 5 used multivariate analyses; the remainder treated gang membership merely as a correlate in descriptive statistics.

A well-established concept that has been overlooked in gang-related research, even in high-income country settings, is adverse childhood experiences (ACEs). Adverse childhood experiences have been primarily investigated in the medical field, since the seminal study by Felitti et al^17^ on their association with the leading causes of death, and encompass child exposure to maltreatment, household dysfunction, and, more recently, community and systemic factors.^17,18^ Adverse childhood experiences have also been linked to criminal behavior, interpersonal violence, criminal justice involvement, and recidivism, suggesting that they are a strong candidate for furthering understanding of gang involvement, which puts individuals at high risk for these outcomes.^19,20,21,22,23^ Although previous studies^24,25^ have examined certain risk factors for gang membership, which can be classified as individual ACEs (eg, hostile family environment), there are currently no studies to our knowledge that have examined the full range of ACEs in relation to gang membership. Crucially, by investigating specific adversities without considering their co-occurrence, the importance of individual ACEs is easily overestimated.^26^ Moreover, without considering how ACEs cluster together, specific constellations of exposures that influence gang membership cannot be identified.

Evidence from Brazil linking ACEs and gang membership is urgently needed for 2 reasons. First, current research on crime and gangs in this high-violence region overwhelmingly focuses on socioeconomic, cultural, and political factors.^27^ This understanding is critical but omits potentially relevant developmental determinants that could also be integrated into an overall crime prevention strategy. Additionally, because of such well-documented socioeconomic and structural drivers of gangs in this context, the hypothesis that ACEs are also important determinants cannot be assumed. Second, Brazil’s response to the gang problem can be largely characterized as *mano dura*, or tough policing and criminal justice strategies, which are considered costly and generally ineffective, with profound social repercussions and human rights violations.^28^ Therefore, there is a critical need to complement existing research on gangs by incorporating data on childhood risk factors into integrated prevention efforts.

In summary, there is a lack of evidence on ACEs and gangs worldwide, and in the Brazilian context, where gangs are understood to be driven by deep socioeconomic forces, the role of ACEs (and, hence, developmental prevention) is unclear. The current study thus aimed to understand the role of ACEs in gang involvement by using longitudinal data from a large Brazilian birth cohort. First, we documented criminal behavior and lifetime criminal justice involvement in young adulthood and their association with gang membership. Second, we examined the associations between ACEs and gang membership, considering ACE exposure individually, cumulatively, and as clusters.

## Methods

### Sample and Procedure

We used data from the 1993 Pelotas Birth Cohort, a prospective, population-based study in Pelotas, Rio Grande do Sul, Brazil. All children born from January 1 to December 31, 1993, were eligible for inclusion. Of the 5265 live births identified through daily hospital visits, 5249 (99.7%) were enrolled in the study. Thus, the study represents the total population of Pelotas, with a high level of socioeconomic diversity, reflecting the significant inequality in Brazil. Maternal skin color, as assessed by the interviewer, was included to examine socioeconomic disparities and categorized as Black, White, and other (no additional details are available to further disaggregate the other category). Methods of follow-up included matching school census registries to birth records, visits to the last known address, obtaining information from other participants, and media campaigns. Assessments were undertaken perinatally and when the children were aged 11 (follow-up rate, 87.5%), 15 (follow-up rate, 85.7%), 18 (follow-up rate, 81.4%), and 22 (follow-up rate, 76.3%) years. Further details on the cohort are documented elsewhere.^29,30^ All participants received information about the study and signed a written informed consent form. This study was approved by the Research Ethics Committee of the Faculty of Medicine at the Federal University of Pelotas. This cohort study followed the Strengthening the Reporting of Observational Studies in Epidemiology (STROBE) reporting guideline.^31^

### Measures and Variables

#### Adverse Childhood Experiences

We assessed 12 lifetime ACEs using child self-reports and maternal reports for children at 11 and/or 15 years of age. Child-reported ACEs included binary indicators of child maltreatment (physical neglect, physical abuse, emotional abuse, and sexual abuse), adapted from the Brazilian version of the Childhood Trauma Questionnaire,^32^ as well as domestic violence, which have demonstrated construct validity^33^ as well as predictive validity for crime outcomes and psychopathology in early adulthood in the same sample.^34,35^ To measure household dysfunction, we included 2 binary indicators of parental divorce and parental death, reported by both informants. Furthermore, maternal mental illness was assessed via the 20-item Self-Report Questionnaire (SRQ-20), a screening questionnaire for common mental disorders in the past month.^36^ The SRQ-20 consists of 20 items coded as yes (1) or no (0), resulting in a total score of 0 to 20, with 0 indicating the absence of symptoms and 20 indicating the presence of symptoms. We used the recommended cutoff score of 8 or higher as a conservative estimate of psychiatric disorders.^37,38^ Ever being separated from parents was reported by the child using a single binary item asking about a permanent change in the primary caregiver that caused feelings of abandonment (as per interview instructions). Finally, we measured community and systemic factors, including self-reported binary items on neighborhood fear and discrimination, and poverty, which was evaluated through changes in family income from participants’ birth to 11 years of age, based on maternal reports. Detailed information on the items, their time points, and sources is presented in eTable 1 in Supplement 1.^34^

#### Gang Membership

Past-year gang membership at 18 years of age was assessed via self-report, using the following item: “In the past 12 months, have you participated in any criminal organisation, faction, or gang?” (in Portuguese: “Nos últimos doze meses, tu participaste de alguma quadrilha, facção ou gangue?”). Responses were coded as yes (1) or no (0).

#### Crime

We assessed past-year crime at 18 and 22 years of age using self-reported items, originally from the Edinburgh Study of Youth Transitions and Crime.^39^ The questionnaire was piloted, adapted, and previously used in several prior studies in this population,^40,41,42^ and has demonstrated concurrent and predictive validity.^42,43^ We created 3 dichotomous crime outcomes at both ages (coded as yes or no): violent, nonviolent, and any crime. Violent crime was defined by 5 items: stole from person with threat or force, assault, carried a weapon for fights or self-defense, used a weapon, and rape. Nonviolent crime included 9 items: stole from shops or stores, damaged property, stole from vehicle, stole vehicle, sold drugs, burgled, sold stolen goods, arson, and stole from person without threat or force.

#### Criminal Justice Involvement

Lifetime involvement in the juvenile and adult criminal justice system was assessed via self-report. Participants were asked at 18 years of age if they had ever been in a youth rehabilitation center and at 18 and 22 years of age if they had ever been detained or arrested. Each of the 3 items was coded as yes or no.

#### Confounders

The following confounding variables of the association between ACEs and gang membership were measured during the perinatal assessment: child sex (coded as male or female) and both maternal and paternal educational level (coded as complete years of formal schooling). Additionally, we used a perinatal health risk score that was previously demonstrated to predict conduct problems and violence in this population^41^ and was also used previously in a study on ACEs.^34^ This score aggregates 6 binary items (range, 0-6): unplanned pregnancy, maternal smoking during pregnancy, maternal alcohol consumption during pregnancy, maternal urinary tract infection during pregnancy, intrauterine growth restriction (defined as <10th percentile or ≥10th percentile for gestational age and gender, based on the Kramer et al^44^ reference curve), and premature birth (<37 weeks’ gestation).

### Statistical Analysis

There were 3794 participants (72.1% of the baseline sample) with complete data on ACEs (eFigure 1 in Supplement 1). Given that ACEs may be missing not at random, imputing these variables could have introduced more bias than using complete case analysis.^45^ We therefore addressed missing data in this sample using multivariate imputation by chained equations with 100 imputed datasets (see the eMethods in Supplement 1 for additional details). Using these imputed data, we first report the proportions of past-year crime and lifetime criminal justice involvement for the total sample and stratified by gang membership. We also compare the odds of these outcomes between gang members and non–gang members. Second, the proportion of youth reporting gang membership is compared between those exposed vs unexposed to each individual ACE.

Third, we examine associations between both individual and cumulative ACEs and gang membership. Sexual abuse was not examined as a single adversity because there were no cases in the gang membership group. The cumulative ACE score is categorized as 0, 1, 2, 3, or 4 or more ACEs, with 0 ACEs as the reference category. In these analyses, proportions are presented with their SE applying Rubin rules, accounting for within-imputation and between-imputation variability. We used a mean bias-reducing scores adjustment as initially described by Kosmidis and Firth^46^ and Firth,^47^ considering the relatively low number of gang members and potential issues of separation and perfect prediction. Effect sizes of regression models are presented as odds ratios (OR) with 95% CIs with the significance level set at a 2-sided *P* < .05. Models were adjusted for child sex, maternal educational level, paternal educational level, and the perinatal health risk score. Details on linearity, independence of errors, and multicollinearity are presented in the eMethods in Supplement 1.

We also examine associations between previously identified latent classes of ACEs and gang membership (additional details in the eMethods in Supplement 1).^34^ Specifically, we compare the odds of gang membership for 2 classes characterized by elevated ACEs (participants exposed to child maltreatment and household challenges and participants exposed to household challenges and social risks), with a low-adversities class. In these analyses, we additionally limited the sample to those with complete data on confounders (N = 3236), which may help support the missing at random assumption. Missing data were addressed using a full information maximum likelihood estimator with robust SEs to account for missing data on gang membership.^48^ The approach by Kosmidis and Firth^46^ and Firth^47^ is not readily available in Mplus, which was used to estimate the latent class model. Therefore, we used binary logistic regression analysis to examine these associations, adjusting for child sex, maternal and paternal educational levels, and the perinatal health risk score. Results are presented as ORs (95% CIs), with the significance level set at a 2-sided *P* < .05. In eTables 4 and 5 in Supplement 1, we also provide unadjusted results for all 3 approaches. All analyses were conducted in RStudio, version 2023.12.0 (R Foundation), except the latent class analysis (LCA), which was performed using Mplus, version 8.1 (Muthén and Muthén). Data analyses were conducted from February to August 2024.

## Results

A total of 3794 participants (1964 [51.8%] female and 1830 [48.2%] male; 703 [18.5%] Black, 2922 [77.0%] White, and 169 [4.5%] other [no additional details are available to further disaggregate the other category]) were included. More sociodemographic characteristics are presented in eTable 2 in Supplement 1, along with comparisons among the baseline sample, the imputation sample, the sample used for LCA, and complete cases. eFigures 1 and 2 in Supplement 1 present the flowchart and missing data patterns, respectively.

### Gang Membership, Crime, and Criminal Justice Involvement

On the basis of the imputed data, 1.6% (SE, 0.2 percentage points) of participants reported gang involvement at 18 years of age. The mean (SE) prevalence of having committed any crime, violent crime, and nonviolent crime at 18 years of age was 17.5% (0.7%), 15.6% (0.7%), and 5.9% (0.4%), respectively, decreasing to 9.8% (0.6%), 8.5% (0.5%), and 3.4% (0.4%), respectively, at 22 years of age. The mean (SE) prevalence of having been in a youth rehabilitation center by 18 years of age was 1.0% (0.2%), while the mean (SE) prevalence of having been detained or arrested reached 3.2% (0.3%) and 4.8% (0.4%) by 18 and 22 years of age, respectively. Gang involvement was associated with all types of crime (violent, nonviolent, and any crime) at both ages, with ORs ranging from 23.32 (95% CI, 11.04-49.23) for any crime to 28.89 (95% CI, 15.94-52.36) for nonviolent crime at 18 years of age and from 7.20 (95% CI, 3.72-13.91) for violent crime to 12.19 (95% CI, 5.76-25.82) for nonviolent crime at 22 years of age (*P* < .001 for all). Furthermore, gang involvement was associated with increased odds of both juvenile and adult criminal justice contact, with ORs ranging from 7.60 (95% CI, 2.41-23.99) for juvenile detention (*P* = .001) to 13.79 (95% CI, 7.28-26.13) for detainment or imprisonment in adulthood (*P* < .001). Table 1 provides full details.

**Table 1.** Descriptive Statistics of Past-Year Crime and Lifetime Criminal Justice Involvement for the Total Sample and Stratified by Past-Year Gang Membership

Crime type	Proportion, mean (SE), %^a^			OR (95% CI)^b^	*P* value
	Total	Non–gang members	Gang members		
**Age of 18 y**					
Any crime	17.5 (0.7)	16.5 (0.7)	82.7 (5.8)	23.32 (11.04-49.23)	<.001
Violent crime	15.6 (0.7)	14.5 (0.6)	82.4 (5.8)	26.80 (12.77-56.24)	<.001
Nonviolent crime	5.9 (0.4)	5.1 (0.4)	60.9 (7.0)	28.89 (15.94-52.36)	<.001
Juvenile detention	1.0 (0.2)	0.9 (0.2)	6.0 (3.9)	7.60 (2.41-23.99)	.001
Detained or imprisoned	3.2 (0.3)	2.8 (0.3)	28.3 (6.3)	13.79 (7.28-26.13)	<.001
**Age of 22 y**					
Any crime	9.8 (0.6)	9.3 (0.5)	43.7 (8.2)	7.61 (3.93-14.72)	<.001
Violent crime	8.5 (0.5)	8.1 (0.5)	38.5 (7.8)	7.20 (3.72-13.91)	<.001
Nonviolent crime	3.4 (0.4)	3.0 (0.3)	27.1 (7.6)	12.19 (5.76-25.82)	<.001
Detained or imprisoned	4.8 (0.4)	4.3 (0.4)	32.1 (7.7)	10.56 (5.17-21.59)	<.001

Abbreviation: OR, odds ratio.

^a^ Based on imputed data (N = 3749).

^b^ Comparing odds of crime and criminal justice involvement outcomes for gang members vs non–gang members.

### ACEs and Gang Membership

Considering each ACE measured in the study, exposed participants had a higher prevalence of gang membership compared with those unexposed, except for neighborhood fear, which showed the opposite pattern. The largest difference related to having been separated from parents, with a mean (SE) of 3.7% (1.3%) reporting gang membership among those exposed and 1.4% (0.2%) among those unexposed, and domestic violence, with 3.6% (1.0%) and 1.3% (0.2%) among those exposed and unexposed, respectively (Table 2). The prevalence of ACEs for the total sample and stratified by gang membership are presented in eTable 3 in Supplement 1.

**Table 2.** Proportions of Past-Year Gang Membership at 18 Years of Age for Gang Members Exposed vs Unexposed to Specific Adverse Childhood Experiences Up to 15 Years of Age

Adverse childhood experience	Proportion, mean (SD), %^a^	
	Unexposed	Exposed
Physical neglect	1.5 (0.2)	1.8 (1.6)
Physical abuse	1.4 (0.2)	3.2 (1.3)
Emotional abuse	1.3 (0.2)	2.5 (0.6)
Domestic violence	1.3 (0.2)	3.6 (1.0)
Maternal mental illness	1.3 (0.2)	2.2 (0.5)
Parental divorce	1.1 (0.2)	2.4 (0.5)
Ever separated from parents	1.4 (0.2)	3.7 (1.3)
Parental death	1.5 (0.2)	2.3 (1.3)
Poverty	1.5 (0.2)	1.9 (0.6)
Discrimination	1.5 (0.2)	2.1 (0.7)
Neighborhood fear	1.7 (0.2)	0.9 (0.5)

^a^ Based on imputed data (N = 3749). Sexual abuse was not examined because there were no cases in the gang membership group.

In adjusted analyses of individual ACEs, physical abuse (OR, 2.76 [95% CI, 1.27-5.98]; *P* = .01), emotional abuse (OR, 2.76 [95% CI, 1.51-5.02]; *P* < .001), domestic violence (OR, 3.39 [95% CI, 1.77-6.48]; *P* < .001), parental divorce (OR, 2.04 [95% CI, 1.17-3.54]; *P* = .01), and ever being separated from parents (OR, 3.13 [95% CI, 1.54-6.37]; *P* = .002) were each associated with increased odds of gang membership. There was no evidence that the remaining ACEs were associated with gang involvement (Table 3). Considering the cumulative ACEs score and risk of gang membership, a dose-response association was observed. The odds of gang involvement increased with the number of ACEs reported, with ORs ranging from 4.50 (95% CI, 1.21-16.77) for 1 ACE to 8.86 (95% CI, 2.24-35.08) for 4 or more ACEs, when compared with no ACE exposure (Table 3).

**Table 3.** Adjusted Associations Between ACEs Up to 15 Years of Age and Past-Year Gang Membership at 18 Years of Age

Analytical approach	OR (95% CI)^a^	*P* value
Singe adversities		
Physical neglect	1.04 (0.29-3.70)	.96
Physical abuse	2.76 (1.27-5.98)	.01
Emotional abuse	2.76 (1.51-5.02)	<.001
Domestic violence	3.39 (1.77-6.48)	<.001
Maternal mental illness	1.52 (0.87-2.67)	.14
Parental divorce	2.04 (1.17-3.54)	.01
Ever separated from parents	3.13 (1.54-6.37)	.002
Parental death	1.57 (0.60-4.12)	.36
Poverty	1.06 (0.54-2.10)	.87
Discrimination	1.54 (0.78-3.05)	.22
Neighborhood fear	0.65 (0.27-1.56)	.34
Cumulative ACE risk score		
0	1.00 [Reference]	.003
1	4.50 (1.21-16.77)	
2	5.07 (1.32-19.49)	
3	7.43 (1.86-29.69)	
≥4	8.86 (2.24-35.08)	

Abbreviations: ACE, adverse childhood experience; OR, odds ratio.

^a^ Based on imputed data (N = 3749). Adjusted for child sex, maternal educational level, paternal educational level, and a cumulative score of biological risk factor. Sexual abuse was not examined because there were no cases in the gang membership group.

Examining ACEs based on LCA, after adjusting for confounders, the child maltreatment and household challenges class had considerably high odds of gang membership compared with the low-adversities class (OR, 7.10; 95% CI, 2.37-21.28). By contrast, the class with exposure to household challenges and social risks had lower odds of gang membership compared with the low-adversities class (OR, 2.28; 95% CI, 0.46-11.25) .

There was some evidence of negative confounding across the 3 approaches (eTables 4 and 5 in Supplement 1). This finding might be because some confounders, such as child sex, were associated in opposite directions with ACEs and gang membership. eTable 6 in Supplement 1 lists the multivariable associations of included confounders.

## Discussion

In this large, prospective, population-based study in Southern Brazil, ACEs reported up to 15 years of age were associated with gang membership at 18 years of age, with experiences of child maltreatment and family conflict being particularly strong risk factors. This study marks the first longitudinal investigation of child risk factors for gang membership in a low- or middle-income country and, to our knowledge, is the first study worldwide to use a multimethod approach to measuring associations between ACEs and gang involvement. Given the profound influences of wider social, economic, institutional, and cultural influences on gangs in Brazil, this new evidence on childhood ACEs holds potentially important implications for complementary approaches to prevention.

In this general population sample, 1.6% of study participants reported past-year gang membership at 18 years of age. Comparisons with other studies are challenging due to differences in how gangs are defined, reference periods, and sample characteristics. For instance, Gatti et al^49^ reported prevalences ranging from less than 1% to more than 16% among youths aged 12 to 15 years across 30 countries. As expected, gang members had increased involvement in both past-year violent and nonviolent crime, concurrently and longitudinally, and were more likely to have had contact with the criminal justice system, complementing the meta-analysis by Pyrooz et al^50^ on gang membership and offending, which primarily included studies from the US and other high-income countries. The decrease in rates of offending from the ages of 18 to 22 years among participants involved in gangs at the age of 18 years may be explained by short duration of gang membership (typically lasting 1-2 years^51^) as well as some desistance from crime after a peak age of 18 years, which is well documented in the literature.^52^

ACEs were associated with increased odds of gang membership individually, cumulatively, and as clusters. Individual ACEs related to child maltreatment and family conflict (physical and emotional abuse, domestic violence, parental divorce, and ever being separated from parents) were particularly associated with gang membership. This conclusion was also supported when examining different patterns of clustering of ACEs; the class characterized by child maltreatment and household challenges was particularly associated with gang membership. Additionally, an increasing number of ACEs was associated with higher odds of gang involvement compared with no exposure to ACEs. These findings align with prior research^14,16^ from high-income countries, highlighting the importance of family risk factors and cumulative burden. The study included a relatively small number of gang members, and CIs for associations with ACEs were notably wide, likely due to the rare outcome. Therefore, although there was strong evidence of associations with ACEs, uncertainty remains about the magnitude of these associations.

### Future Research

Future studies need to confirm the magnitude of associations between ACEs and gang membership in this and other contexts as well as investigate other potential differential associations among types of ACEs, for example, between ACEs classified as imposing threat vs deprivation.^53^ In addition, gang researchers should draw further on insights from more than 2 decades of ACE research. It seems particularly advisable to use multiple approaches to analyze ACEs, given that each approach has distinct limitations. These limitations include confounding issues with a single adversity approach, the assumption of equal weighting across ACEs in cumulative risk scores, and generalizability concerns regarding empirically driven methods, such as LCA.^26^ Finally, it will be crucial to examine mechanisms linking ACEs with gang involvement, especially those that can be targeted by interventions. For example, in a sample of more than 100 000 juvenile offenders in Florida, the association between cumulative ACE exposure and gang membership was largely mediated by substance abuse and difficult temperament.^54^

### Implications for Public Health

Although gangs are primarily addressed in secondary and tertiary prevention,^55^ the current findings emphasize a critical primary prevention perspective. They support calls to eradicate violence against children, including interventions to reduce harsh and abusive parenting. Parenting interventions focused on child maltreatment have proven effective in reducing antisocial behavior and delinquency.^56,57^ Crime and gang involvement are closely intertwined, and given the shared risk factors between them,^14^ existing programs could be adapted to meet the needs of youth at risk for gang affiliation. However, preventive and treatment efforts certainly should not be limited to the family environment but also target broader social inequalities and other community and macro influences, which may directly cause gang membership or act as precursors to family risk factors.

### Limitations

This study has some limitations. Attrition in this longitudinal study involved some sociodemographic patterning, which we tried to address with imputing missing variables. Although the small number of gang members was examined using regression methods for rare outcomes, this approach was not applicable for analyses of latent classes and gang membership, necessitating caution when interpreting these results. Gang membership was measured using a single item; additional questions about the nature and extent of gang involvement would have been beneficial. Although some associations may have been subject to shared rater bias (eg, gang members may have exaggerated their involvement in criminal behavior to reinforce their identity), this is less concerning for the association between ACEs and gang membership because ACEs were based on a combination of child and maternal reports. Some potential confounding variables were missing from the analyses, including, for example, parental experiences of child maltreatment.^58^

## Conclusions

This cohort study found that gang members were at very high risk of crime, as expected. ACEs, especially those related to child maltreatment and family conflict, were associated with later gang involvement when examined individually, cumulatively, and as clusters. Researchers of gang membership may want to integrate the ACE framework and leverage its 2 decades of progress into uncovering early causes of gang membership toward effective primary prevention.

## Notes

Supplement 1. **eMethods.** Expanded Notes on Study Methods **eTable 1.** Items Used to Measure Adverse Childhood Experiences, Their Time Points, and the Informant Used **eFigure 1.** Flow Chart **eFigure 2.** Missing Data Patterns **eTable 2.** Comparison of Sociodemographic Characteristics Across Baseline, Imputed, LCA, and Complete Case Samples **eTable 3.** Prevalence of Adverse Childhood Experiences for the Total Sample and Stratified by Gang Membership **eTable 4.** Unadjusted Associations Between Adverse Childhood Experiences Up to Age 15 and Past-Year Gang Membership at Age 18 **eTable 5.** Unadjusted Associations Between Latent Classes of Adverse Childhood Experiences Up to Age 15 and Past-Year Gang Membership at Age 18 **eTable 6.** Multivariable Associations of Included Confounders With Adverse Childhood Experiences and Gang Membership
eReferences

Supplement 2.
Data Sharing Statement
