# Supplementary material for: Prevalence and Risk Factors of Gang Membership in a Brazilian Birth Cohort
Source: JAMA Netw Open. 2024 Oct 21;7(10):e2440393. doi: 10.1001/jamanetworkopen.2024.40393 (PMC11581666; doi:10.1001/jamanetworkopen.2024.40393)
Supplement: Supplement 1. — eMethods. Expanded Notes on Study Methods eTable 1. Items Used to Measure Adverse Childhood Experiences, Their Time Points, and the Informant Used eFigure 1. Flow Chart eFigure 2. Missing Data Patterns eTable 2. Comparison of Sociodemographic Characteristics Across Baseline, Imputed, LCA, and Complete Case Samples eTable 3. Prevalence of Adverse Childhood Experiences for the Total Sample and Stratified by Gang Membership eTable 4. Unadjusted Associations Between Adverse Childhood Experiences Up to Age 15 and Past-Year Gang Membership at Age 18 eTable 5. Unadjusted Associations Between Latent Classes of Adverse Childhood Experiences Up to Age 15 and Past-Year Gang Membership at Age 18 eTable 6. Multivariable Associations of Included Confounders With Adverse Childhood Experiences and Gang Membership eReferences [file jamanetwopen-e2440393-s001.pdf]

Supplementary Online Content

Bauer A, Martins RC, Hammerton G, et al. Prevalence and risk factors of gang membership in a Brazilian birth cohort. *JAMA Netw Open*. 2024;7(10):e2440393. doi:10.1001/jamanetworkopen.2024.40393

eMethods. Expanded Notes on Study Methods

eTable 1. Items Used to Measure Adverse Childhood Experiences, Their Time Points, and the Informant Used

eFigure 1. Flowchart

eFigure 2. Missing Data Patterns

eTable 2. Comparison of Sociodemographic Characteristics Across Baseline, Imputed, LCA, and Complete Case Samples

eTable 3. Prevalence of Adverse Childhood Experiences for the Total Sample and Stratified by Gang Membership

eTable 4. Unadjusted Associations Between Adverse Childhood Experiences Up to Age 15 and Past-Year Gang Membership at Age 18

eTable 5. Unadjusted Associations Between Latent Classes of Adverse Childhood Experiences Up to Age 15 and Past-Year Gang Membership at Age 18

eTable 6. Multivariable Associations of Included Confounders With Adverse Childhood Experiences and Gang Membership

eReferences

This supplementary material has been provided by the authors to give readers additional information about their work.

© 2024 Bauer A et al. *JAMA Network Open.*

eMethods. Expanded Notes on Study Methods

Multiple imputation

When examining the associations of adverse childhood experiences (ACEs), crime, and criminal justice involvement with gang membership, we addressed missingness using multivariate imputation by chained equations (MICE) with 100 imputed data sets. MICE specifies the imputation model on a variable-by-variable basis, without assuming a joint distribution of all variables, as required by other approaches to imputing multivariate data. Considering the rare outcome of gang membership and the use of composite measures – specifically 'any crime' and the cumulative ACE risk score – we developed four distinct imputation models. These models were tailored to: cumulative risk and any crime; (2) cumulative risk and violent/non-violent crime; (3) individual ACEs and any crime; and (4) individual ACEs and violent/non-violent crime. In each model, all study variables were specified to impute each other. We also used auxiliary variables, such as deviant peer associations and conduct problems at age 11, to improve the imputation of crime outcomes and criminal justice involvement at age 18. The convergence of each model was verified, imputed values were checked for plausibility, and Monte Carlo standard errors were assessed to ensure the integrity of our statistical analyses.[1]

Latent class analysis

In a previous study investigating the association between ACEs and crime outcomes, we identified three distinct subgroups of participants based on ACE exposure patterns using latent class analysis (LCA). To select the optimal class model, we compared 1-6 class solutions, considering model fit indices, entropy, sample size of the smallest class, and interpretability of each class.[2] When examining the associations between the derived classes and outcomes, we used a 1-step approach, which is recommended for models with relatively low entropy.[3] Further details on the model fit indices, entropy, class counts/proportions across all class solutions, class characteristics of the 3-class model, and their associations with crime outcomes are detailed in another publication.[4]

Assumptions of logistic regression

Assumptions were tested for the models examining adjusted associations between individual ACEs and gang membership, based on non-imputed data. Confounding variables included child sex, maternal education, paternal education, and a perinatal health risk score. Since the main predictor variables were binary, the linearity assumption was not further investigated. The assumption of independence of errors was considered fulfilled, as the cases in this study are not related to one another. Finally, multicollinearity was examined using the variance inflation factor (VIF) and tolerance statistics, with values of ≥ 10 and < 0.1, respectively, being considered problematic. VIF values for child sex, maternal education, paternal education, and the perinatal health risk score ranged between 1.00 and 1.05, 1.43 and 1.50, 1.41 and 1.47, and 1.03 and 1.05, respectively, indicating minimal multicollinearity. Similarly, the tolerance statistics for child sex (0.95-1.00), maternal education (0.66-0.71), paternal education (0.68-0.71), and the perinatal health risk score (0.96-0.97) showed no evidence of multicollinearity.

© 2024 Bauer A et al. *JAMA Network Open.*

eTable 1 Items used to measure adverse childhood experiences, their time points, and the informant used

Adverse childhood experiences	Item(s)	Age(s)	Informant
Physical neglect	*Have you ever not had enough food at home or had to wear dirty/worn clothes because you had no others?*	15	Child
Physical abuse[a]	*In the past 6 months, has an adult of your family or someone who was looking after you hit you in a way that left you hurt or bruised?*	11, 15	Child
Emotional abuse[b]	*Have you ever thought or felt that your parents did not want you to have been born? Have you ever thought or felt that someone in your family hates you?*	15	Child
Sexual abuse	*Has anyone ever tried to do sexual things to you against your will, threatening or hurting you?*	15	Child
Domestic violence	*Have there ever been fights with physical assault in your household between adults or has an adult ever assaulted a child or adolescent?*	15	Child
Maternal mental illness	A score of ≥ 8 on the SRQ-20, as recommend by Mari & Williams, 1986[5]	11	Mother
Parental divorce[b]	*Are your parents separated?*<br>*Have you and the natural father of your child ever get divorced?*	15	Child<br>Mother
Ever separated from parents	*Have you ever been separated from your parents to be taken care of by someone else?*	15	Child
Parental death[a]	*Is your natural mother/father alive?*<br>*Is the natural mother/father alive?*	11, 15	Child<br>Mother
Poverty	Measured as change in family income, coded as 'always poor' (lower tercile of family income at both time points) and 'not always poor' (middle/upper tercile at either time point)	birth, 11	Mother
Discrimination	*Since last month, have you felt discriminated or disadvantaged because of your skin colour/race, religion, wealth/poverty, illness/physical disability?*	11	Child
Neighbourhood fear	*Have you ever been in fear of living in your neighbourhood?*	11	Child

Note. [a] = If answered affirmatively at either time point. [b] = If either item was answered affirmatively. SRQ = Self-Report Questionnaire.

© 2024 Bauer A et al. *JAMA Network Open.*

eFigure 1 Flow chart

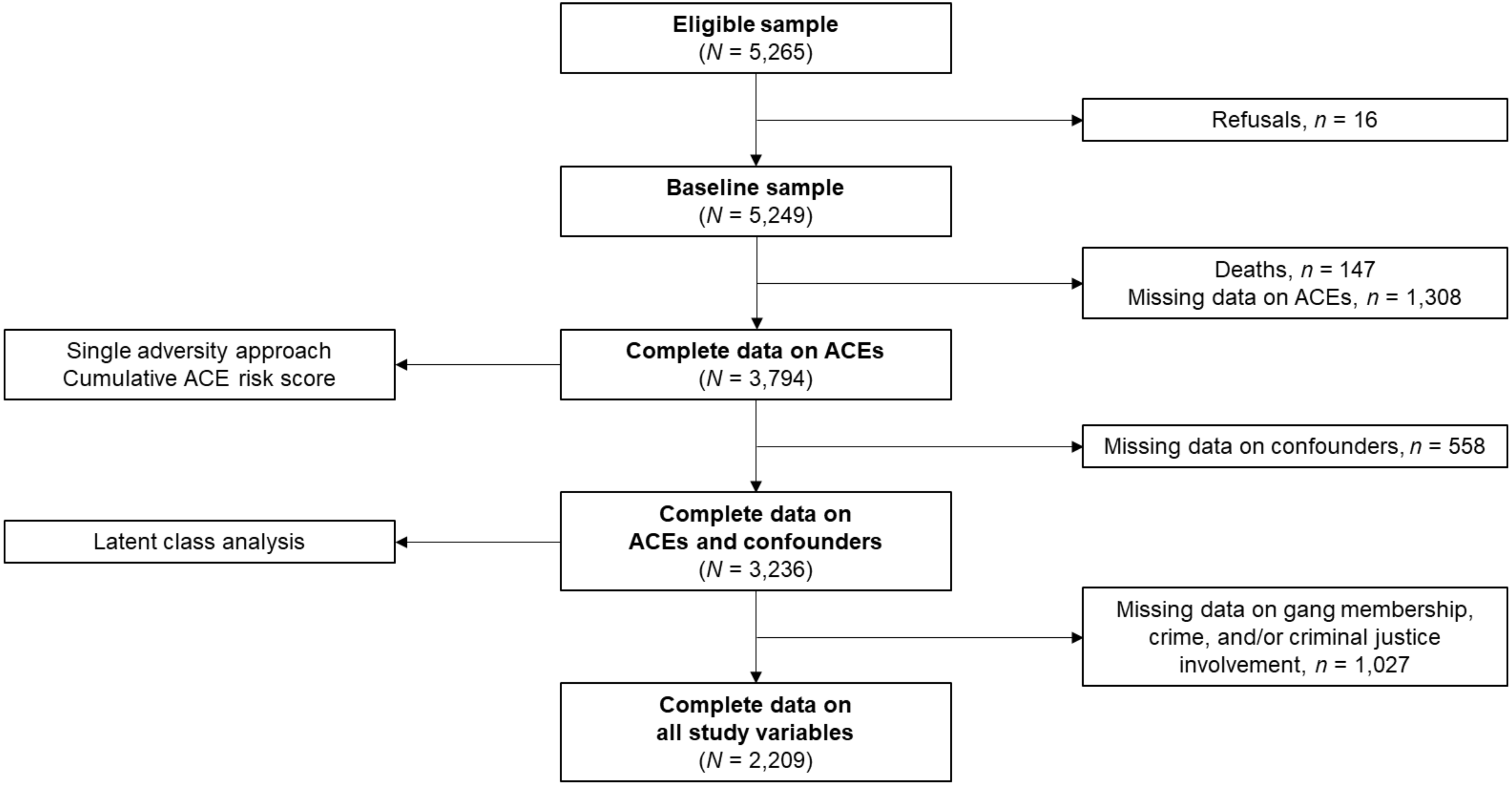

Note. ACEs = Adverse childhood experiences.

© 2024 Bauer A et al. *JAMA Network Open.*

eFigure 2 Missing data patterns

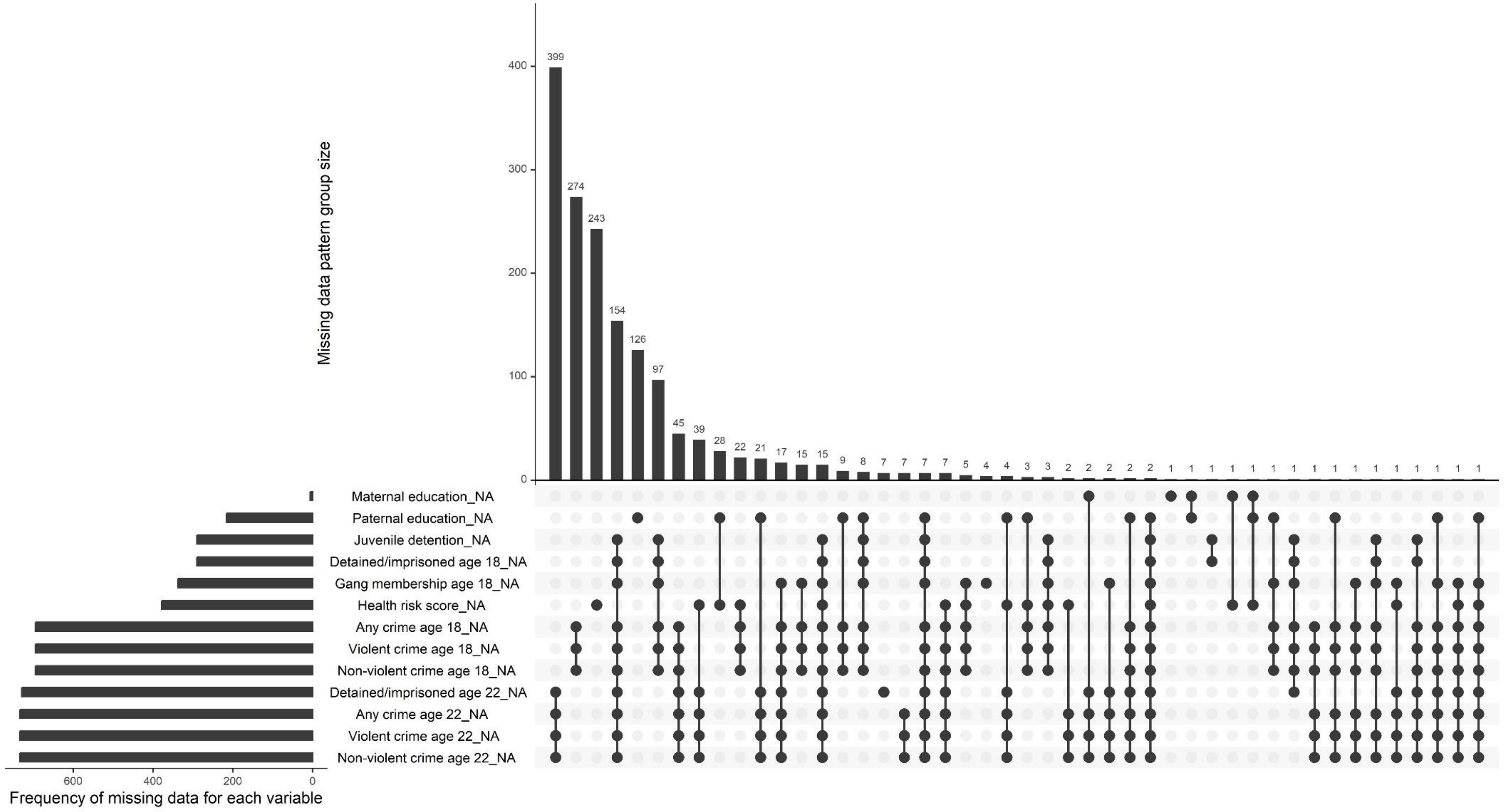

Note. Based on complete data for adverse childhood experiences items, N = 3,794. The bars on the left represent the frequency of missing data for each variable, while the linked/single dots represent distinct missing data combinations, with the bars on top indicating their frequencies. Ordered in descending order by missing data pattern group size.

© 2024 Bauer A et al. *JAMA Network Open.*

eTable 2 Comparison of sociodemographic characteristics across baseline, imputed, LCA, and complete case samples

	**Baseline sample** (*N* = 5,249) % (*n*) or Mean (SD)	**Imputation sample** (*N* = 3,794) % (*n*) or Mean (SD)	**LCA sample** (*N* = 3,236) % (*n*) or Mean (SD)	**Complete cases** (*N* = 2,209) % (*n*) or Mean (SD)
**Child sex**				
Male	49.6 (2603)	48.2 (1830)	48.2 (1561)	46.0 (1017)
**Maternal education**	6.7 (3.6)	6.8 (3.5)	7.0 (3.5)	7.1 (3.5)
**Paternal education**	6.8 (3.5)	6.8 (3.5)	6.9 (3.5)	7.0 (3.5)
**Maternal age**	26.0 (6.4)	26.1 (6.4)	26.2 (6.3)	26.3 (6.3)
**Maternal smoking**				
Yes	33.4 (1752)	33.0 (1252)	31.8 (1029)	30.8 (681)
**Maternal alcohol consumption**				
Yes	5.1 (267)	5.4 (203)	5.0 (163)	4.6 (102)
**Mother living with partner**				
No	12.4 (649)	10.9 (412)	8.5 (274)	9.2 (204)
**Maternal skin colour**[a]				
Black	18.2 (955)	18.5 (703)	17.2 (556)	17.2 (380)
White	77.3 (4058)	77.0 (2922)	78.9 (2552)	78.7 (1738)
Other	4.5 (234)	4.5 (169)	4.0 (128)	4.1 (91)
**Adverse childhood experiences**				
Physical neglect	4.8 (198)	4.6 (173)	4.1 (133)	3.9 (86)
Physical abuse	7.1 (296)	6.9 (262)	6.7 (217)	6.6 (145)
Emotional abuse	20.4 (845)	19.9 (756)	19.4 (628)	19.5 (430)
Sexual abuse	1.5 (61)	1.4 (54)	1.4 (44)	1.5 (33)
Domestic violence	10.4 (433)	10.3 (391)	10.2 (329)	10.6 (234)
Maternal mental illness	30.9 (1360)	30.0 (1139)	29.4 (950)	29.1 (642)
Parental divorce	35.7 (1545)	35.4 (1341)	33.9 (1098)	34.8 (768)
Ever separated from parents	8.8 (368)	8.1 (307)	7.8 (252)	7.6 (168)
Parental death	7.1 (307)	6.4 (241)	5.9 (192)	5.3 (118)
Poverty	20.8 (890)	19.9 (754)	18.4 (594)	17.8 (393)
Discrimination	16.4 (726)	15.5 (589)	15.1 (489)	14.3 (316)
Neighbourhood fear	16.0 (709)	16.2 (614)	16.2 (524)	16.2 (358)
**Gang membership**				
Yes	1.4 (58)	1.5 (50)	1.4 (41)	1.3 (28)
**Crime (age 18 years)**				
Any crime	17.8 (644)	17.5 (541)	17.3 (456)	16.1 (356)
Violent crime	15.6 (566)	15.5 (481)	15.3 (403)	14.2 (313)
Non-violent crime	6.1 (222)	5.9 (183)	6.0 (157)	5.4 (120)

© 2024 Bauer A et al. *JAMA Network Open.*

Juvenile detention	1.2 (50)	0.9 (33)	0.9 (27)	0.8 (18)
Detained/imprisoned	3.5 (143)	3.1 (110)	3.2 (94)	2.6 (58)
**Crime (age 22 years)**				
Any crime	9.7 (346)	9.2 (282)	9.3 (243)	9.8 (217)
Violent crime	8.2 (295)	7.9 (241)	7.9 (205)	8.3 (183)
Non-violent crime	3.3 (119)	2.9 (90)	3.0 (79)	3.3 (73)
Detained/imprisoned	4.5 (163)	4.2 (128)	4.0 (104)	4.3 (94)

Note. Baseline sample = All available data. Imputation sample = Complete data on adverse childhood experiences. Latent class analysis (LCA) sample = Complete data on adverse childhood experiences and confounders. [a] = Assessed by the interviewer.

© 2024 Bauer A et al. *JAMA Network Open.*

eTable 3 Prevalence of adverse childhood experiences for the total sample and stratified by gang membership

Analytical approach	Prevalence % (SE)		
	Total sample	Non-gang members	Gang members
**Singe adversities**			
Physical neglect	4.6 (0.3)	4.5 (0.3)	5.3 (4.4)
Physical abuse	6.9 (0.4)	6.8 (0.4)	14.1 (5.1)
Emotional abuse	19.9 (0.6)	19.7 (0.7)	31.4 (6.5)
Sexual abuse[a]	1.4 (0.2)	NA	NA
Domestic violence	10.3 (0.5)	10.1 (0.5)	23.5 (6.0)
Maternal mental illness	30.0 (0.7)	29.8 (0.7)	42.7 (6.9)
Parental divorce	35.3 (0.8)	35.1 (0.8)	53.3 (7.0)
Ever separated from parents	8.1 (0.4)	7.9 (0.4)	19.3 (5.8)
Parental death	6.4 (0.4)	6.3 (0.4)	9.5 (4.8)
Poverty	19.9 (0.6)	19.8 (0.7)	24.6 (6.2)
Discrimination	15.5 (0.6)	15.4 (0.6)	21.2 (6.1)
Neighbourhood fear	16.2 (0.6)	16.3 (0.6)	9.8 (4.5)
**Cumulative ACE risk score**			
0	23.1 (0.7)	23.4 (0.7)	4.2 (3.7)
1	29.0 (0.7)	29.0 (0.7)	29.0 (6.4)
2	21.1 (0.7)	21.1 (0.7)	23.5 (6.2)
3	13.6 (0.6)	13.5 (0.6)	20.9 (6.1)
4+	13.2 (0.5)	13.1 (0.6)	22.0 (6.0)

Note. Based on imputed data (*N* = 3,749). [a] = Sexual abuse was not examined, because there were no cases in the gang membership group.

© 2024 Bauer A et al. *JAMA Network Open.*

eTable 4 Unadjusted associations between adverse childhood experiences up to age 15 and past-year gang membership at age 18

Analytical approach	Gang membership	
	OR (95% CI)	*p*-value
**Singe adversities**		
Physical neglect	1.35 (0.38-4.77)	0.64
Physical abuse	2.37 (1.09-5.13)	0.03
Emotional abuse	1.89 (1.05-3.41)	0.03
Sexual abuse[a]	NA	NA
Domestic violence	2.80 (1.48-5.31)	0.002
Maternal mental illness	1.76 (1.01-3.06)	0.04
Parental divorce	2.11 (1.21-3.67)	0.008
Ever separated from parents	2.86 (1.41-5.82)	0.004
Parental death	1.68 (0.64-4.42)	0.29
Poverty	1.35 (0.71-2.57)	0.35
Discrimination	1.52 (0.76-3.02)	0.23
Neighbourhood fear	0.60 (0.25-1.45)	0.25
**Cumulative ACE risk score**		
0	*Ref*	
1	4.71 (1.25-17.72)	
2	5.29 (1.37-20.43)	0.003
3	7.39 (1.85-29.45)	
4+	8.03 (2.05-31.47)	

Note. Based on imputed data (*N* = 3,749). OR (95% CI) = Odds ratio (95% confidence interval). ACE = Adverse childhood experiences. [a] = Sexual abuse was not examined, because there were no cases in the gang membership group.

© 2024 Bauer A et al. *JAMA Network Open.*

eTable 5 Unadjusted associations between latent classes adverse childhood experiences up to age 15 and past-year gang membership at age 18

**Latent classes**	**Gang membership**	
	OR (95% CI)	*p*-value
Low adversities	*Ref*	
Child maltreatment / household challenges	5.11 (1.69-15.49)	< 0.001
Household challenges / socials risks	2.87 (0.63-12.99)	

Note. Based on imputed data (*N* = 3,749). OR (95% CI) = Odds ratio (95% confidence interval). ACE = Adverse childhood experiences.

© 2024 Bauer A et al. *JAMA Network Open.*

eTable 6 Multivariable associations of included confounders with adverse childhood experiences and gang membership

	Child sex ('male')		Maternal education		Paternal education		Biological risk score	
	OR (95% CI)	*p*-value	OR (95% CI)	*p*-value	OR (95% CI)	*p*-value	OR (95% CI)	*p*-value
**Exposures**								
Physical neglect	1.36 (1.00-1.85)	0.05	0.87 (0.82-0.93)	< .001	0.96 (0.90-1.02)	0.19	1.29 (1.11-1.50)	0.001
Physical abuse	0.70 (0.54-0.91)	0.007	1.02 (0.98-1.07)	0.32	0.95 (0.90-0.99)	0.02	1.03 (0.90-1.16)	0.70
Emotional abuse	0.40 (0.33-0.47)	< .001	0.95 (0.93-0.98)	0.002	1.01 (0.98-1.05)	0.34	1.09 (1.00-1.18)	0.04
Domestic violence	0.58 (0.47-0.72)	< .001	0.99 (0.95-1.03)	0.48	1.01 (0.97-1.05)	0.65	1.24 (1.12-1.36)	< .001
Maternal mental illness	1.11 (0.96-1.28)	0.14	0.88 (0.86-0.91)	< .001	1.00 (0.97-1.02)	0.85	1.23 (1.14-1.32)	< .001
Parental divorce	0.98 (0.86-1.13)	0.82	0.97 (0.95-1.00)	0.03	1.01 (0.99-1.04)	0.40	1.18 (1.10-1.26)	< .001
Ever separated from parents	0.79 (0.62-1.00)	0.05	0.98 (0.94-1.03)	0.46	0.98 (0.94-1.03)	0.45	1.05 (0.93-1.18)	0.41
Parental death	1.00 (0.77-1.30)	0.99	0.92 (0.88-0.97)	0.002	0.94 (0.90-0.99)	0.02	1.01 (0.88-1.15)	0.91
Poverty	1.22 (1.02-1.45)	0.03	0.79 (0.76-0.82)	< .001	0.85 (0.82-0.88)	< .001	1.13 (1.04-1.23)	0.005
Discrimination	0.84 (0.71-1.01)	0.06	0.96 (0.93-0.99)	0.02	0.97 (0.94-1.00)	0.09	1.13 (1.04-1.23)	0.005
Neighbourhood fear	0.75 (0.63-0.89)	0.001	1.00 (0.97-1.03)	0.99	1.01 (0.97-1.04)	0.74	1.12 (1.03-1.22)	0.010
**Outcome**								
Gang membership	5.50 (2.64-11.44)	< .001	0.92 (0.83-1.02)	0.13	1.01 (0.91-1.12)	0.82	1.12 (0.86-1.47)	0.40

Note. Based on imputed data (*N* = 3,749). Each association between a specific confounder and the exposure(s) and outcome is adjusted for the remaining confounders.

© 2024 Bauer A et al. *JAMA Network Open.*

eReferences

1. White IR, Royston P, Wood AM. Multiple imputation using chained equations: issues and guidance for practice. *Statistics in medicine.* 2010;30(4):377-399. doi: 10.1002/sim.4067
2. Wickrama KA, Lee TK, O'Neal CW, Lorenz FO. *Higher-order growth curves and mixture modeling with Mplus: a practical guide.* New York, NY: Routledge; 2016.
3. Bakk Z, Tekle FB, Vermunt JK. Estimating the association between latent class membership and external variables using bias-adjusted three-step approaches. *Sociol Methodol.* 2013;43(1):272-311. doi: 10.1177/0081175012470644
4. Bauer A, Martins RC, Hammerton G, et al. Adverse childhood experiences and crime outcomes in early adulthood: a multi-method approach in a Brazilian birth cohort. *Psychiatry Res.* 2024;334. doi: 10.1016/j.psychres.2024.115809
5. Mari JJ, Williams P. A validity study of a psychiatric screening questionnaire (SRQ-20) in primary care in the city of Sao Paulo. *Br J Psychiatry.* 1986;148:23-26. doi: 10.1192/bjp.148.1.23

© 2024 Bauer A et al. *JAMA Network Open.*
