# Supplementary material for: Prevalence and Risk Factors of Gang Membership in a Brazilian Birth Cohort
Source: JAMA Netw Open. 2024 Oct 21;7(10):e2440393. doi: 10.1001/jamanetworkopen.2024.40393 (PMC11581666; doi:10.1001/jamanetworkopen.2024.40393)
Supplement: Supplement 2. — Data Sharing Statement [file jamanetwopen-e2440393-s002.pdf]

Data Sharing Statement

Bauer. Prevalence and Risk Factors of Gang Membership in a Brazilian Birth Cohort. *JAMA Netw Open*. Published October 21, 2024. doi:10.1001/jamanetworkopen.2024.40393

Data
Data available: No

Additional Information
Explanation for why data not available: Applications to use the data can be made by contacting the researchers of the 1993 cohort (see http://www.epidemio-ufpel.org.br/site/content/faculty/ for a list of key faculty members) and completing the application form (http://www.epidemio-ufpel.org.br/site/content/studies/formularios.php). A list of administered questionnaires at each timepoint can be accessed online (http://www.epidemio-ufpel.org.br/site/content/coorte_1993-en/questionnaires.php). Researchers with successful applications will receive a dataset including the requested variables and unique participant IDs.
